# Determining the Actual Zinc and Iron Intakes in Breastfed Infants: Protocol for a Longitudinal Observational Study

**DOI:** 10.2196/19119

**Published:** 2020-11-06

**Authors:** Oraporn Dumrongwongsiri, Pattanee Winichagoon, Nalinee Chongviriyaphan, Umaporn Suthutvoravut, Veit Grote, Berthold Koletzko

**Affiliations:** 1 Center for International Health Lugwig-Maximilians-Universitaet Munich Munich Germany; 2 Department of Pediatrics Faculty of Medicine Ramathibodi Hospital Mahidol University Bangkok Thailand; 3 Community/International Nutrition Institute of Nutrition Mahidol University Nakhon Pathom Thailand; 4 Department of Pediatrics Dr von Hauner Children's Hospital University Hospital, Lugwig-Maximilians-Universitaet Munich Munich Germany

**Keywords:** breastfeeding, zinc, iron, zinc deficiency, iron deficiency, deuterium oxide dose-to-mother technique, infant, baby, diet, protocol, prediction, women, growth

## Abstract

**Background:**

Zinc and iron deficiencies among breastfed infants during the first 6 months of life have been reported in previous studies. The amounts of zinc and iron intakes from breast milk are factors that contribute to the zinc and iron status of breastfed infants.

**Objective:**

This study aims to quantitatively determine zinc and iron intakes by breastfed infants during the first 4 months of life and to investigate the factors that predict zinc and iron status in breastfed infants.

**Methods:**

Pregnant women at 28 to 34 weeks of gestation were enrolled. Zinc and iron status during pregnancy was assessed. At delivery, cord blood was analyzed for zinc and iron levels. Participants and their babies were followed at 2 and 4 months postpartum. Maternal dietary intakes and anthropometric measurements were performed. The amount of breast milk intake was assessed using the deuterium oxide dose-to-mother technique. Breast milk samples were collected for determination of zinc and iron levels. The amount of zinc and iron consumed by infants was calculated. Zinc and iron status was determined in mothers and infants at 4 months postpartum.

**Results:**

A total of 120 pregnant women were enrolled, and 80 mother-infant pairs completed the study (56 provided full breastfeeding, and 24 provided breast milk with infant formula). All data are being managed and cleaned. Statistical analysis will be done.

**Conclusions:**

This study will provide information on zinc and iron intakes in exclusively breastfed infants during the first 4 months of life and explore predictive factors and the possible association of zinc and iron intakes with infant growth and nutrient status.

**International Registered Report Identifier (IRRID):**

DERR1-10.2196/19119

## Introduction

Micronutrients are essential for infant growth and development. During the first 6 months of life, infants obtain micronutrients from breast milk if they are exclusively breastfed, as recommended by the World Health Organization (WHO) [1]. In addition to micronutrients provided by breast milk, infants use body stores of micronutrients deposited during pregnancy.

Zinc and iron are particularly important micronutrients for infants. Some previous studies have shown that zinc and iron deficiencies are associated with delayed infant growth and development, especially when such deficiencies occur during the early period of life [2-4]. Several reports have shown zinc and iron deficiencies in a high proportion of infants younger than 6 months, and breastfeeding was found to be the associated factor. A study in 2007 in the northeast area of Thailand reported that 50.8% of breastfed infants aged 4 months had a zinc deficiency [5]. When comparing the prevalence of zinc deficiency by feeding types, zinc deficiency was more prevalent among 4- to 6-month old breastfed infants compared with formula-fed infants (14.9% and 5.3% among breastfed and formula-fed infants, respectively) [6]. Regarding iron deficiency, the prevalence of iron deficiency among breastfed infants was found to be higher than among infants fed with formula in several studies [7,8]. A study on the iron status of infants in Bangkok showed that the prevalence of iron deficiency anemia in breastfed infants aged 1 year was 25.7%, which was higher than in formula-fed infants (2.7%) [9]. The percentage of infants with iron deficiency anemia was 4 times higher in 6-month-old compared with 4-month-old infants (26.1% vs 5.7%, respectively), as described in a cohort study of iron status in breastfed infants [10]. Breastfeeding duration was found to be associated with a higher prevalence of iron deficiency [11] and low serum ferritin or other iron markers among infants and children [12,13].

The zinc and iron status of breastfed infants during breastfeeding is associated with several factors. The amount of iron storage during intrauterine life, zinc and iron intakes, and physiologic requirement are proposed to be the factors determining zinc and iron status of breastfed infants [14]. Infants’ iron storage depends on maternal nutrient status during pregnancy and can be observed in cord blood levels [15,16]. Daily zinc and iron intakes of exclusively breastfed infants come from zinc and iron in breast milk. Naturally, micronutrient concentrations in breast milk are not constant but dynamically change during lactation. Zinc and iron concentrations in breast milk are high during early lactation and gradually decline thereafter [17-19]. The amounts of zinc and iron in breast milk consumed by infants after 6 months are lower than the estimated daily requirements [20,21]. The majority of zinc and iron intakes in infants during this period need to be provided by complementary foods.

Several hypotheses have been proposed to explain the causes of zinc and iron deficiencies among breastfed infants during the exclusive breastfeeding period. Low micronutrient concentrations in breast milk have been proposed as a factor associated with nutrient deficiency in breastfed infants. Stronger evidence was shown in the case of zinc deficiency compared with iron deficiency [22]. Recent studies have attempted to explore the factors that might be associated with the low micronutrient concentrations in breast milk. There have been reports on genetic variation of zinc transporters resulting in the difference in breast milk zinc concentrations [23,24]. Some studies have reported that socioeconomic status, maternal dietary intake, maternal anthropometric parameters, micronutrient status, and maternal age are associated with zinc and iron concentrations in breast milk [6,25,26]. However, many studies did not confirm these associations [27,28].

Breast milk provides complete nutrition to infants during the first 6 months of life, but zinc and iron deficiencies occur among breastfed infants. While zinc and iron levels in breast milk have been determined and reported in several studies, they do not directly reflect the zinc and iron intake amounts in breastfed infants. The data on breast milk volume taken by infants and the nutrient levels in breast milk better demonstrate the amounts of zinc and iron taken by breastfed infants. However, the measurement of breast milk volume consumed by infants can be challenging. Traditional assessments using the test-weighing method or the measurement of expressed breast milk have considerable inaccuracies. Stable isotope measurement of breast milk intake with the protocol established by the International Atomic Energy Agency (IAEA) is the most accurate method to quantify the infant‘s breast milk intake [29].

Zinc and iron intake from breast milk is one of the factors determining the zinc and iron status of breastfed infants. The data on nutrient intakes will provide more information regarding the zinc and iron status of breastfed infants and may lead to the prevention of nutrient deficiencies. Our study aims to quantify zinc and iron intakes by measuring micronutrient levels in breast milk and assessing breast milk volume intake by breastfed infants using the deuterium oxide dose-to-mother technique.

## Methods

### Recruitment

**Figure 1:**
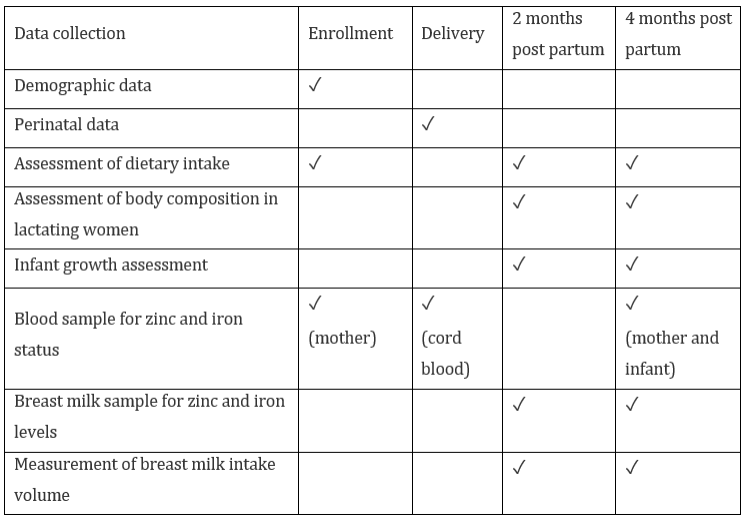
Data collected at each visit in the study.

This is a prospective descriptive study at the Faculty of Medicine Ramathibodi Hospital, Mahidol University, Bangkok, Thailand. The study protocol was approved by the human research ethics committee of the Faculty of Medicine Ramathibodi Hospital, Mahidol University (ID 03-60-31) and the ethical committee of Ludwig Maximillian Universitaet, Munich (Project No. 18-015). Pregnant women visiting the antenatal care (ANC) clinic at Ramathibodi Hospital at 28 to 34 weeks of gestation were eligible for enrollment. The enrollment was performed at the ANC clinic when the pregnant women attended the education class during their second trimester. Inclusion criteria were healthy pregnant women who planned to deliver their babies at Ramathibodi Hospital, intended to breastfeed their babies at least 4 months, lived in the Bangkok metropolitan area, and provided written informed consent. Pregnant women who carried twin or triplet pregnancies or who had any contraindication for breastfeeding were excluded. Women and their babies were followed until 4 months postpartum. Each participant was invited to 4 visits (ie, at enrollment, at delivery, and at 2- and 4-month postpartum). The details of the data collection are summarized in Figure 1. During the study, women who were unwilling to participate in the study, stopped breastfeeding, moved to another province, or had babies with chronic diseases or serious illness were excluded from continuing study participation. The recruitment period was from March 2018 to September 2019.

### Sample Size Calculation

Sample size calculation was based on the reported mean zinc intake of breastfed infants in a study by Krebs et al [30], which was 1.00 (SD 0.43) mg/day. The sample size was determined using the 1-sample *t* test for mean formula, as follows:


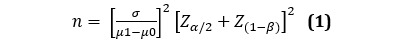


Using the assumption that this study will provide a difference in the mean of 0.15 from the previous study, and given a significance level of .05 and power of 0.8, the calculated sample size of this study was 64:


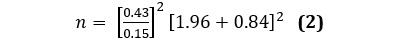


n=64

The calculated sample size of this study was 64. We estimated that the dropout rate would be up to 30%. Therefore, the calculated sample size was 100 participants:


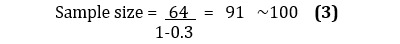


### Data Collection

#### Demographic Data and Antenatal Data

Demographic data, including maternal age, existing diseases, education level, and socioeconomic status, were obtained by interviewing participants. Antenatal data were retrospectively reviewed from medical records and the maternal pregnancy handbook. Data collected during pregnancy included prepregnant weight and BMI, weight gain during pregnancy, parity, investigations during antenatal care (every pregnant woman had a blood test for anemia and serologic screening during their first visit to the ANC clinic and some had an oral glucose tolerance test to screen for gestational diabetes, depending on clinical indication), and complications during pregnancy (ie, gestational diabetes, preeclampsia, and others).

#### Perinatal Data

The investigators visited the participants who delivered their babies at Ramathibodi Hospital at the postpartum ward. Mothers received routine postpartum care and education from the ward staff. Data regarding mode of delivery, delivery complications, and perinatal complications of infants were collected. Infant anthropometric data, including birth weight, length, and head circumference, were routinely measured by nurses in the labor room.

#### Anthropometric Assessment of Lactating Women and Infants

To determine the nutritional status of lactating women and infants, anthropometric measurements were performed at the 2- and 4-month postpartum visits. For lactating woman, weight was measured to the nearest 0.1 kg using a digital scale, and height was measured to the nearest 0.1 cm using a height scale while the woman was standing upright without shoes or hair ornaments. Weight, BMI, fat mass, skeletal muscle mass, and visceral fat area were measured using a body composition analyzer (InBody 720; InBody Co).

For the infant, weight was measured to the nearest 10 grams using a digital baby scale, and recumbent length was measured to the nearest 0.1 cm using a wooden board with a sliding foot piece. Head circumference was determined using a nonstretchable measuring tape. The occipitofrontal circumference was measured twice; the greater value was used to represent the baby’s head circumference. Weight, length, and head circumference were calculated to *z* score for age and sex, according to the WHO growth chart from the WHO Anthro calculator. In addition to the anthropometric measurements, the weight and length gain of the infants was calculated to determine the growth rate during the first 4 months.

#### Assessment of Maternal Dietary Intakes

Dietary intake was important during pregnancy and lactation. There are a lot of factors influencing maternal food intake during these periods, such as beliefs and traditions, lifestyle, anxiety, socioeconomic status, and family support. We assessed maternal dietary intakes during pregnancy (at enrollment) and lactation (at 2- and 4-month postpartum) using 3 dietary intake assessment tools, namely a 24-hour food recall, the food frequency questionnaire (FFQ), and a 3-day prospective dietary record. The FFQ was constructed to determine zinc and iron intake with common foods eaten by Thai people. At participant visits, dietary history (24-hour food recall and FFQ) was recorded by a skilled dietitian or nutritionist. A 3-day food record form was then handed to the participant to complete at home within 2 weeks after the visit. They were asked to send the food record back to the researcher by mail or to bring it back at the next visit. The amounts of nutrient intake, including energy, protein, zinc, and iron, were analyzed using INMUCAL software version 4.0 (Institute of Nutrition, Mahidol University), which is the largest database of nutrients in Thai foods.

#### Collection and Analysis of Blood Samples

Blood samples for determining zinc and iron status were collected. Maternal blood samples were collected from an antecubital vein during pregnancy (at enrollment) and lactation (at 4-month postpartum). At delivery, cord blood samples were collected right after cord cutting from the umbilical cord on the placental side. Infant venous blood samples were collected at the age of 4 months.

Blood samples were immediately centrifuged to separate plasma and kept frozen at –80 °C. All containers used for sample collection were washed with a nitric acid solution and deionized water in order to avoid contamination with micronutrients from the environment. Zinc concentration was analyzed using flame atomic absorption spectrophotometry (GBC Avanta S; GBC Scientific Equipment). Serum ferritin and complete blood count were analyzed using chemiluminescence (automated) and electrical impedance, respectively, at the Department of Pathology, Faculty of Medicine Ramathibodi Hospital. Remaining plasma samples were kept for further analysis.

#### Collection and Analysis of Breast Milk

Each lactating woman was asked to collect a breast milk sample at the 2- and 4-month visits. Breast milk samples were collected from one breast by an electrical milk pump. The participant was asked to express breast milk until the breast was empty. The breast milk sample was then evenly mixed, and 15 mL of the breast milk sample was collected for analysis. The remaining breast milk was kept in the milk storage bag and returned to the participant for feeding her infant. The milk samples were kept at –80 °C within 4 hours from the time of collection. All the equipment and containers used in breast milk collection had been washed with a nitric acid solution and deionized water to avoid micronutrient contamination from the environment and were sterilized before use. A separate aliquot of 5 mL of the evenly mixed breast milk sample was transferred for metabolomics analyses.

Zinc and iron levels in the breast milk were analyzed using inductively coupled plasma optical emission spectrometry (ICP-OES). Prior to analysis by ICP-OES, the breast milk sample was digested using nitric acid in a closed vessel under microwave radiation.

#### Measurement of Breast Milk Volume Intake by Infants Using Deuterium Oxide Dose-to-Mother Method

The breast milk volume was assessed at 2- and 4-month postpartum. In general practice, there are two methods of giving breast milk to infants: providing breastfeeding at the breast or expressing breast milk via bottle-feeding. We used different methods to assess breast milk volume from different feeding practices.

Among mothers who provide breastfeeding at the breast, breast milk intake was assessed using the deuterium oxide dose-to-mother technique, strictly following the protocol to assess breast milk intake proposed by the IAEA [29]. Deuterium is a stable (nonradioactive) isotope of hydrogen that is metabolized in the body in the same way as water. Therefore, deuterium oxide is eliminated from the body in urine, saliva, sweat, and human milk.

**Figure 2:**
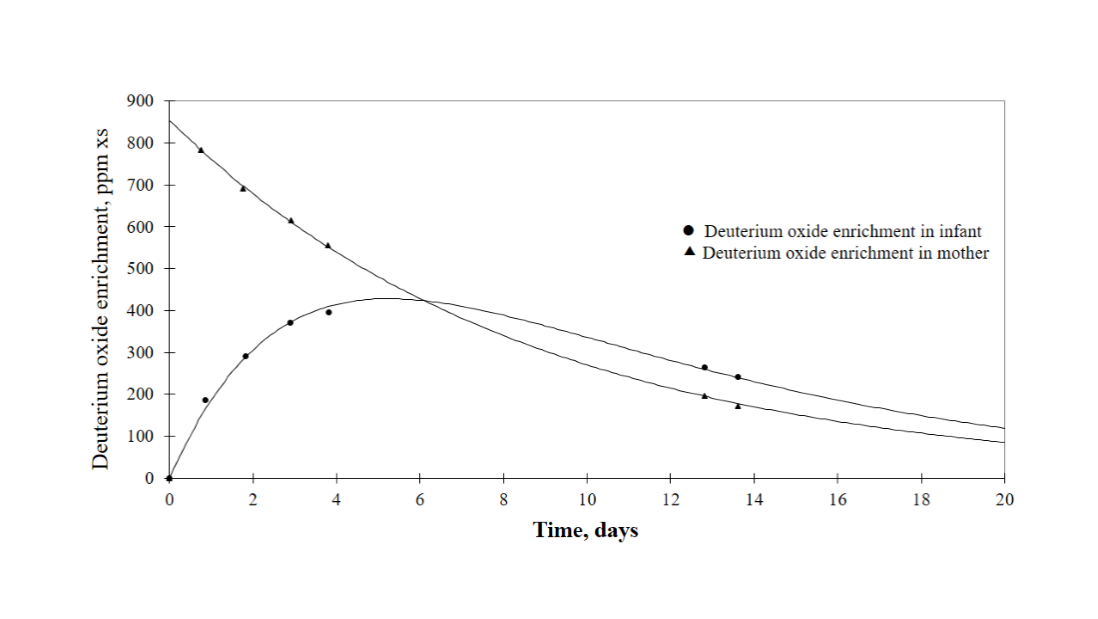
Deuterium oxide enrichment from the deuterium oxide dose-to-mother technique to measure breast milk intake shows the disappearance of deuterium oxide from the mother's body and the presence of deuterium oxide in the infant. xs: excess.

The principle of the deuterium oxide dose-to-mother technique for measuring breast milk volume is to track the disappearance of deuterium oxide from the maternal body and the presence of deuterium oxide in the infant (Figure 2). Lactating women were given a drink of 30 g of deuterium-labeled water. Saliva samples (2 mL per sample) were collected from mothers and infants to monitor deuterium oxide levels. According to the IAEA protocol, saliva samples were collected at 7 time points: at baseline (day 0) before giving the deuterium-labeled water to the mother and at days 1, 2, 3, 4, 13, and 14 after the dose of deuterium. All samples were collected by the same researchers, both at the hospital (on day 0) and during home visits. Deuterium oxide levels were analyzed and breast milk volumes were calculated using the equation based on the principles of volume distribution [29].

## Results

Study enrollment took place from March 2018 to September 2019. A total of 120 pregnant women participated in this study. There were 3 participants who delivered their babies in other hospitals and were excluded from the study. Among the 117 participants, 56 women provided breastfeeding to their babies and completed the study at 4-month postpartum; they were classified as the breastfeeding group. A total of 24 women could not adequately provide breastfeeding to their babies and gave the infants some infant formula. However, they completed the study and were classified as the mixed-feeding group. A total of 37 women were excluded from the study (18 stopped breastfeeding before 4 months, 12 moved to another province, and 7 were unwilling to continue the study). All data are being managed and cleaned. Statistical analysis will be done.

## Discussion

### Expected Outcome

This study will provide information on zinc and iron intakes from breast milk in breastfed infants during the first 4 months of life. It may demonstrate the association of zinc and iron intakes with the growth and nutrient status of infants. As this study follows the participants from pregnancy to lactation, the data may provide information about the impact of intrauterine nutrition on the nutrient status of infants after birth. The data regarding dietary intake and nutrient status of mothers during both the pregnancy and lactation period will be provided and the relationship between maternal and infant nutritional status may be demonstrated.

### Significance of the Study

This study will provide informative data on zinc and iron intakes by breastfed infants. These data will provide scientific knowledge and might contribute to determining the daily dietary zinc and iron requirements for infants during the first 6 months. Moreover, these data may be useful in devising strategies for preventing zinc and iron deficiency in breastfed infants. As the study will also provide information on the levels of zinc and iron in breast milk and their associations with the dietary intake and micronutrient status of lactating women, these data will have advantages for nutritional promotion during lactation.

## Abbreviations

ANC: antenatal care
FFQ: food frequency questionnaire
IAEA: International Atomic Energy Agency
ICP-OES: inductively coupled plasma optical emission spectrometry
WHO: World Health Organization
